# Personalized beyond Precision: Designing Unbiased Gold Standards to Improve Single-Subject Studies of Personal Genome Dynamics from Gene Products

**DOI:** 10.3390/jpm11010024

**Published:** 2020-12-31

**Authors:** Samir Rachid Zaim, Colleen Kenost, Hao Helen Zhang, Yves A. Lussier

**Affiliations:** 1 Center for Biomedical Informatics & Biostatistics of the University of Arizona Health Sciences, The University of Arizona, 1230 N. Cherry Avenue, Tucson, AZ 85721, USA; samirrachidzaim@arizona.edu (S.R.Z.); ckenost@arizona.edu (C.K.); 2 Graduate Interdisciplinary Program in Statistics of the University of Arizona, The University of Arizona, 617 N. Santa Rita Avenue, P.O. Box 210089, Tucson, AZ 85721, USA; hzhang@math.arizona.edu; 3 Department of Mathematics, The University of Arizona, 617 N. Santa Rita Avenue, P.O. Box 210089, Tucson, AZ 85721, USA; 4 Department of Medicine, College of Medicine Tucson, 1501 N. Campbell Avenue, P.O. Box 245017, Tucson, AZ 85724, USA; 5 Arizona Cancer Center, 1501 N. Campbell Avenue, P.O. Box 245017, Tucson, AZ 85724, USA

**Keywords:** single-subject studies, personalized medicine, precision medicine, reference standards, gold standards, biomarkers, open-source

## Abstract

**Background:** Developing patient-centric baseline standards that enable the detection of clinically significant outlier gene products on a genome-scale remains an unaddressed challenge required for advancing personalized medicine beyond the small pools of subjects implied by “precision medicine”. This manuscript proposes a novel approach for reference standard development to evaluate the accuracy of single-subject analyses of transcriptomes and offers extensions into proteomes and metabolomes. In evaluation frameworks for which the distributional assumptions of statistical testing imperfectly model genome dynamics of gene products, artefacts and biases are confounded with authentic signals. Model confirmation biases escalate when studies use the same analytical methods in the discovery sets and reference standards. In such studies, replicated biases are confounded with measures of accuracy. We hypothesized that developing method-agnostic reference standards would reduce such replication biases. We propose to evaluate discovery methods with a reference standard derived from a consensus of analytical methods distinct from the discovery one to minimize statistical artefact biases. Our methods involve thresholding effect-size and expression-level filtering of results to improve consensus between analytical methods. We developed and released an R package “referenceNof1” to facilitate the construction of robust reference standards. **Results:** Since RNA-Seq data analysis methods often rely on binomial and negative binomial assumptions to non-parametric analyses, the differences create statistical noise and make the reference standards method dependent. In our experimental design, the accuracy of 30 distinct combinations of fold changes (FC) and expression counts (hereinafter “expression”) were determined for five types of RNA analyses in two different datasets. This design was applied to two distinct datasets: Breast cancer cell lines and a yeast study with isogenic biological replicates in two experimental conditions. Furthermore, the reference standard (RS) comprised all RNA analytical methods with the exception of the method testing accuracy. To mitigate biases towards a specific analytical method, the pairwise Jaccard Concordance Index between observed results of distinct analytical methods were calculated for optimization. Optimization through thresholding effect-size and expression-level reduced the greatest discordances between distinct methods’ analytical results and resulted in a 65% increase in concordance. **Conclusions:** We have demonstrated that comparing accuracies of different single-subject analysis methods for clinical optimization in transcriptomics requires a new evaluation framework. Reliable and robust reference standards, independent of the evaluated method, can be obtained under a limited number of parameter combinations: Fold change (FC) ranges thresholds, expression level cutoffs, and exclusion of the tested method from the RS development process. When applying anticonservative reference standard frameworks (e.g., using the same method for RS development and prediction), most of the concordant signal between prediction and Gold Standard (GS) cannot be confirmed by other methods, which we conclude as biased results. Statistical tests to determine DEGs from a single-subject study generate many biased results requiring subsequent filtering to increase reliability. Conventional single-subject studies pertain to one or a few patient’s measures over time and require a substantial conceptual framework extension to address the numerous measures in genome-wide analyses of gene products. The proposed *referenceNof1* framework addresses some of the inherent challenges for improving transcriptome scale single-subject analyses by providing a robust approach to constructing reference standards.

## 1. Introduction

From both biological and statistical standpoints, reproducibility and accuracy of results are crucial to clinical utility of genome-wide Omics studies. A 2016 survey by *Nature* [1] indicated that 70% of researchers failed to replicate other scientists’ studies, with more than half failing to replicate their own. While the accuracy and reproducibility of an Omics signal in multi-subject studies can be assessed by comparing subjects in distinct datasets, evaluating the accuracy of a single-subject study (SSS) remains challenging. In principle, conventional statistics deriving dispersion parameters (e.g., variance) across samples can be applied to single-subject studies using multiple repeated measures in each compared condition (e.g., *t*-test) or many measures over time (e.g., time series) [2,3]. However, this strategy is often prohibitively expensive, wastes valuable clinical specimens, and is rate-limiting. The foundation for single-subject studies [4,5] highlights the challenges and issues associated with inferential statistics on cohorts of size N = 1. Beyond the multiple repeated measures paradigm of conventional statistics, we and others have proposed new analytical methods designed to identify an effect size and statistical significance for a subject from an Omics sample per condition without replicates [6,7,8,9] (in this study specifically with transcriptomics). A reference standard consisting of the other subjects’ genomes is sufficient to qualify the frequency of a genetic variant or mutation in static DNA. However, when this strategy is applied to proteins or transcripts, it does not inform on the differences observed between an individual’s gene products expression and that of a group. Are these differences attributable to a normal physiological adaption or to a pathological response to environmental factors unique to this individual (e.g., a combination of medication)? Furthermore, how can studies best capture these differences? This manuscript presents an alternative approach to constructing method-independent reference standards that address inherent challenges in transcriptome scale single-subject studies. A proposed framework improves upon the evaluation of software tools and algorithms for differential gene expression in one subject between two sampling conditions, in absence of replicate measures per condition. Such single-subject study Omics designs are more affordable and practical for clinical settings than repeated measures in one condition. Generally, they provide a more interpretable effect size and p-value at a single subject than comparing an individual against a cohort. We contrast and compare this new evaluation framework in transcriptomics to previous ones in terms of the accuracy of results beyond the previously proposed “naïve replication” and quantify biases stemming from previous evaluations’ anticonservative assumptions frameworks.

In large-scale biological data science studies, “gold standards” produced via biological validation is rate-limiting and generally unfeasible at the Omics scale. Data scientists address this limitation with computational “reference standards” as a proxy for conventional biological gold standards. The most rigorous reference standards employ (i) independent analytics and (ii) independent samples (datasets) from predictions. However, these two conditions are not always feasible in single-subject studies. Furthermore, most approaches generating reference standards from an Omics dataset rely heavily on *p*-values, despite recommendations from statistician scholars for effect-size informed approaches to address the limitations of null-hypothesis significance testing [10,11]. We synthesize and incorporate these notions into a set of standard operating procedures for the development of reliable reference standards in transcriptomes, as a foundation for evaluating big data science reproducibility studies.

This manuscript focuses on improving the accuracy of single-subject studies evaluations, beyond “naïve reproducibility” of results and other biases described in Table 1. In a prior study of 5 distinct RNA analysis methods in multiple isogenic datasets [12], we described a new method that combines the inconsistent signal between analytical methods that the original study did not address [13]. This inconsistency (arising from distributional differences) required methods, such as DESeq [14], to impose a false discovery rate (FDR) cutoff of 0.001 to detect ~3000 DEGs, while DEGseq [15] required a cutoff of FDR < 3.6 × 10^−12^ for the same number of DEGs, with 2039 overlapping transcripts. Conversely, we also found that applying the same FDR cutoff (i.e., 0.001) resulted in methods producing various predictions (i.e., 3200 vs. ~9000 with approximately 3000 overlapping transcripts, leaving ~6000 transcripts with a conflicting, unaddressed signal). Anticonservative isomorphic evaluations (Table 1) have been the conventional standard for evaluating DEG analytics in isogenic conditions (e.g., cell lines or inbred animal models), the closest datasets to single-subject studies [13,16]. Such evaluations propose a naïve replication of results using the anticonservative assumption that the same DEG analytics can be employed to create the reference standard and the predictions. In a prior study, we constructed an ensemble learner [12] to develop reference standards, where the ensemble approach resolves conflicting biomarker prediction, uses no statistical assumptions, and removes anti-conservative isomorphic evaluations. Our prior study demonstrated that in situations comprising high technical noise, an ensemble learner maximizes the stability of a reference standard and the DEG predictions [12]. However, they increase the “black-box” aspect of the data analysis and muddle its interpretability.

**Figure 1:**
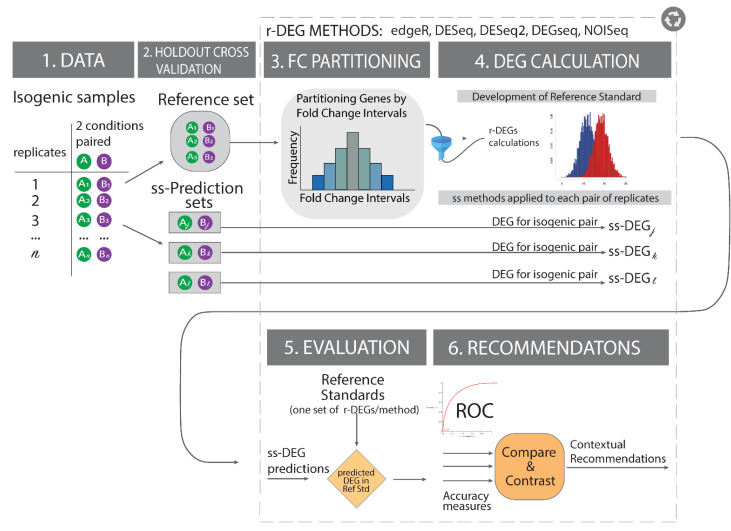
**Reference Standard Construction Study Overview.** In isogenic paired samples from historical cell lines datasets exposed to two conditions (condition A in green and B in purple), we first split the paired stimulus-control data into non-overlapping reference and prediction sets. To maximize biological interpretability and relevance, we run all differentially expressed genes (DEG) calculations and organize the results by fold change regions and conduct all evaluations. We introduce the effect-size (Fold change) analysis into the study to mitigate noisy results (i.e., low p-values with negligible effect sizes) while maximizing biological interpretability and quantify this improvement via accuracy measures (i.e., Area under the ROC curve) on an exemplar study. **Notation:** ss = single-subject, ss-DEG*i* = differentially expressed genes in a single subject “*i*”, conditions: A or B, A*_k_* = gene product expression of gene “*k*” in condition A; B*_k_* = gene product expression of gene “*k*” in condition B.

In principle, the reference standard should be independent from the predicted biological signal to evaluate an analytical method. This requires independent datasets for calculating and evaluating the prediction. We sought to improve the evaluation of single-subject studies of transcriptomics-scale gene products by generating unbiased reference standards. We focused on one framework of single-subject studies: Those with two transcriptomics-scale measures (one per condition) in one subject, designed to determined altered gene products using the subject as their own control. We hypothesized that these unbiased reference standards could be achieved by: (i) Using distinct analytical methods against than the one being evaluated to avoid analytical biases, and (ii) selecting the most concordant results between multiple analytical methods according to ranges of fold change expression between two conditions and expression count cutoffs. We propose a framework, *referenceNof1*, to resolve the challenges highlighted in Table 1, offering an alternate, yet related, evaluation framework for improving the data quality in the reference standard construction. The framework is presented in Figure 1. We demonstrate the *referenceNof1* method accuracy with transcriptome simulations and historical transcriptome data (Section 2). Section 3 discusses the implications and limitations of the current approaches, while Section 4 details the data and materials and formally introduces the *referenceNof1* algorithm. Section 5 concludes the study. The *referenceNof1* software is released as an R package.

## 2. Methods and Materials

The study design is illustrated in Figure 1, and the following subsections detail the datasets and materials used throughout the study. To improve the state of the art in building reference standards, the study is designed around isogenic datasets, addressing the issues in Table 1.

### 2.1. Datasets

In this study, two different isogenic datasets with biological replicates were used to evaluate the construction of reference standards, shown in Table 2. Isogenic datasets comprising more than 5 repeated RNAseq measures on different biological sample (not technical replicates) in two experimental conditions (>110 samples) are difficult to find in the literature and were best designed to test our hypothesis. We found two that had previously served as reference standards in conventional RNASeq analyses requiring repeated measures, which we use here without repeated measures for the discovery set (1 sample per condition), and with repeated measures for the validation set.

The first is an MCF7 breast cancer dataset that contains replicated gene expression data in isogenic conditions with 7 human biological replicates of MCF7 cells, which were either treated with 10 nM 17β-estradiol (E2) or cultured as unstimulated controls [13]. The data contain replicates at various read depths with all analyses being conducted using the 30M read replicates, which are available open source in the Gene Expression Omnibus repository [17] under the “GSE51403” GEO tag. Normalized and preprocessed data were downloaded on 21 January 2018. The data were used as obtained with no additional preprocessing steps to conduct the reproducibility analyses (preprocessing details and correction details can be found in the original publication) [13], and we randomly selected four biological replicates (“565–576”,”564–572”,”566–570”,”562–574”) to construct the reference standard, while the remaining three (“563–577”,”568–575”,”569–571”) were used to evaluate how well the DEG methods recapture the signal under different reference standard settings. The second dataset [16] is a yeast study with biological replicates comprised of 48 wild-type (BY4741 strain, WT) or **Δ**snf2 mutant biological yeast replicates (*Saccharomyces cerevisiae)*. These included a total of 7126 measured genes, and we replicated following the author’s data preprocessing framework and conducted our studies using their suggested 42 WT and 44 **Δ**snf2 ‘clean’ replicates. The preprocessed and normalized data were downloaded as prepared by the original authors from their GitHub (Data downloaded from on 27th August 2018. https://github.com/bartongroup/profDGE48) repository.

### 2.2. Software Environment

All analyses in this study were conducted in the R programming language, using R 3.5.0 [18] on a 2017 MacBook Pro under the macOS High Sierra (10.13.6) OS system. All code and analyses are freely available in our GitHub repository.

### 2.3. Differential Expression Software Tools

To evaluate the robustness and reproducibility of various differential gene expression analyses techniques, we evaluated 5 different cohort-based (cb) RNA-seq tools, found in Supplementary Materials Table S1 [14,15,19,20,21]. Since the study was designed to evaluate the robustness of reference standards, we omitted the single-subject analytics included in our prior study (i.e., Mixture Models [7] and iDEG [9]), as these methods are designed to predict DEGs in isogenic settings, two conditions without replicates (TWCR) design, rather than produce reference standards in replicated settings. We omitted GFOLD [22] and similar ranking-based methods as they did not include p-values to establish consistent cutoffs in our experimental design. Supplementary Table S1 provides the individual parameter settings for each method.

### 2.4. Building Effect-Size-Informed Reference Standards

We hypothesize that low effect sizes (i.e., low FC) introduce statistical noise into the reference standard construction in isogenic conditions, possibly creating biases when using only p-value informed DEGs. Therefore, to test this hypothesis, we first construct a reference standard for each method using all the data, and then degrade the dataset by filtering out genes with effect sizes, in an increasing fashion and evaluate the strength of the agreement across them. Thus, if we use fold change (FC) as a proxy for effect size (as calculated by Equation (1)),

Fold Change of gene *k* = *A_k_*/*B_k_* (1)

where *A_k_* and *B_k_* are the expression of gene product *k* in condition *A* and *B* (Figure 1). The experimental design was comprised of constructing the reference standard across different levels of fold change (FC) and evaluating the concordance as a consequence of the effect size filter. Since we do not distinguish between up- and down-regulated genes, for down-regulated genes, we take their reciprocal, (FC^−1^ *=* 1/FC) when we filter for FC thresholds. In the manuscript and figures, we will use FC to represent both upregulated genes fold changes and 1/FC for downregulated genes.

### 2.5. Low Expression Pre-Filtering

We hypothesize that low gene expression introduces statistical instability when identifying differentially expressed genes. A common preprocessing approach in differential gene expression is prefiltering genes [23,24,25] with low expression as this may increase the power of the subsequent statistical test. We extend this work into precision medicine by examining the effects of gene pre-filtering in constructing reference standards in isogenic conditions.

### 2.6. Experimental Design

To evaluate the power of combining prefiltering genes based on their effect size (fold change) and expression level, we considered an array of low-expression cutoffs and fold-change regions (see Table 3) and selected genes in these windows.

**Figure 2:**
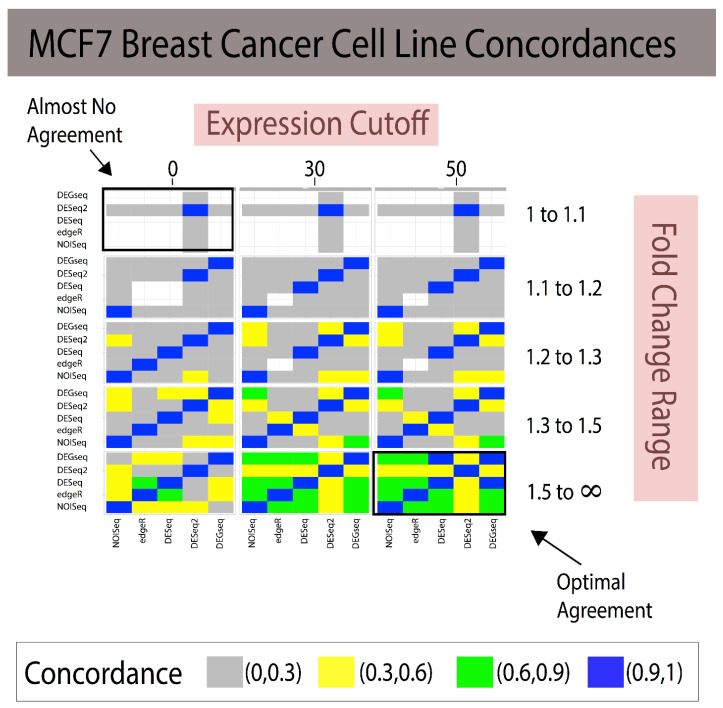
Combining fold change and expression level filtering leads to robust, method-agnostic reference standards for single-subject studies in breast cancer. The grid of heatmaps illustrates that as low-expression genes with small fold changes across individuals are filtered out, the reference standards constructed agree increasingly more with one another. In the bottom right, most methods attain a 100% concordance with one another, providing a reliable gold standard. Expression cutoffs are applied to genes whose average counts across samples fall under the threshold. White cells indicate that no predictions were made, and therefore the Jaccard Index cannot be calculated. Note: When the JI cannot be calculated due to the lack of transcripts, the color of the rectangle is white; in addition, FC ranges are symmetric 1/1.1 to 1 and 1 to 1.1, 1/1.2 to 1 and 1 to 1.2, etc. (Legend: In the manuscript and figure, we represent FC for upregulated genes and 1/FC for downregulated genes).

**Figure 3:**
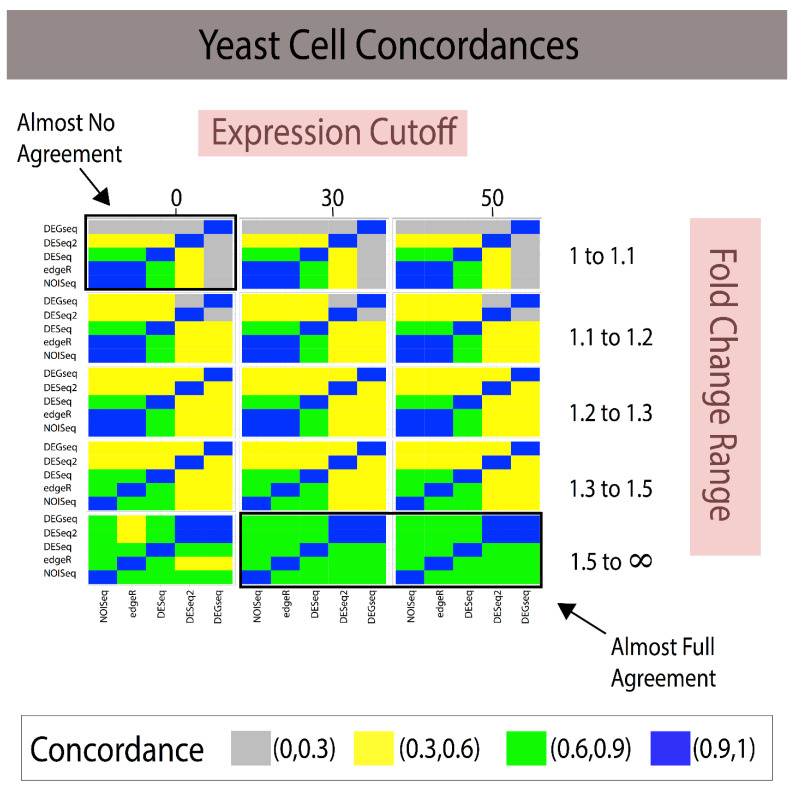
Combining fold change and expression level filtering leads to robust, method-agnostic reference standards for single-subject studies in yeast. The grid of heatmaps illustrates that as low-expression genes with small fold changes across individuals are filtered out, the reference standards constructed provide higher concordance with one another. Given the larger number of replicates in yeast, it may be that less rigid filters are required to produce reliable, concordant reference standards. Expression cutoffs in average counts across samples. White cells indicate that no predictions were made, and therefore the Jaccard Index cannot be calculated. Note: When the Jaccard Index cannot be calculated due to the lack of transcripts, the color of the rectangle is white; in addition, FC ranges are symmetrical 1/1.1 to 1 and 1–1.1, 1/1.2–1 and 1–1.2, etc. (Legend: In the manuscript and figure, we represent FC for upregulated genes and 1/FC for downregulated genes).

We then used these selected genes to construct reference standards for all the methods presented in Supplementary Table S1 and evaluated their concordances. To evaluate the results of the different experimental runs, heatmaps were used to visually compare their concordances across different experimental runs (see Figure 2 and Figure 3) and the Jaccard Index to numerically evaluate the agreement between them, where the Jaccard Index is given by Equation (2).

(2) $$\mathrm{Jaccard}\;\mathrm{Index}\;=\;\frac{\left|M\cap N\right|}{\left|M\cup N\right|}$$

In our study, these metrics translate to the similarity and dissimilarity between the DEG calls between method A and method B (i.e., between edgeR and DESeq), which quantifies the biological signal reproducibility between analytical approaches in our reference standard construction study.

### 2.7. Optimization of a Reference Standard Using Maximum Jaccard Index Concordance

The parameters in the experimental design were constructed to identify regions in which the Jaccard Index was maximized. In this grid search, each set of parameter combinations results in a Concordance Matrix of Jaccard distances (Supplementary Table S2). From this Jaccard matrix, each method’s median Jaccard Index can be calculated (i.e., NOISeq’s median concordance is 0.71 while DEGseq’s is 0.21). One can summarize this information to understand which methods agree with one another and which differ. To construct the most robust reference standard, one needs to identify the optimal parameter combination (fold change and expression-level thresholds) that maximizes the Jaccard Index across all reference standards. Therefore, we constructed an R package, *referenceNof1*, to enable bioinformaticians to construct the optimal reference standard. If a user inputs a vector of effect size windows, a vector of minimum value expression cutoffs, and a desired level of concordance, the *referenceNof1* calculates all pairwise Jaccard similarity indices for all parameter combinations and identifies the minimum parameter combination that attains the desired pairwise concordance across all techniques. This algorithm is formalized in Algorithm 1:
**Algorithm 1. The *referenceNof1* algorithm pseudocode to construct an optimized and unbiased reference standard.**
VariableDescriptionInputsFCA list of fold change thresholdsCutoffsA list of expression thresholdsTargetA minimum median Jaccard index to attain **Steps****For** genes in region $R_{i}$ using Cutoff_i_ and FC_i_, **do****For** method ∈{edgeR, DEGseq, DESeq2, NOISeq}
**do**Identify set of differentially expressed genes**End for**Calculate pairwise Jaccard Index (JI) for all (M,N) pairs of methods:$JI_{\left(M,N\right),R_{i}}=\frac{\left|M\cup N\right|}{\left|M\cap N\right|}$Calculate median Jaccard Index$JI_{R_{i,Med}}=\;median\left(JI_{\left(M,N\right),R_{i}}\right)$**If** ($JI_{R_{i,Med}}\geq$ Target)**Return** Cutoff_*_ = Cutoff_i_, FC_*_ = FC_i_**Else**Update FC, Cutoff parameters**End for****If (Target attained)****Return** Cutoff_*_, FC_*_**Else****Print** No threshold achieved target Jaccard IndexThe *referenceNof1* algorithm requires a user to input the FC and expression cutoff filters for it to then identify the optimal region for producing the reference standard. For each pair of FC-region and expression cutoff combination, it calculates each method’s list of differentially expressed genes (DEGs), and then for each DEG list it calculates the Jaccard Index as a set-theoretic pairwise similarity measure. After calculating all pairwise Jaccard indices, it calculates the median for each region. If a parameter combination attains the desired median Jaccard Index, an early stopping rule is implemented, and the optimal parameter combination is returned. Otherwise, it continues the search until the target Jaccard Index is attained or the search through the parameter space is complete. (Legend: In the manuscript and figure, we represent FC for upregulated genes and 1/FC for downregulated genes.”)

### 2.8. Comparing the Proposed Reference Standard Optimization with a Single Heteromorphic One

To illustrate the benefits of creating more robust reference standards, we conducted an exemplar study using DESeq. As shown in Figure 1, the analysis consisted of constructing a reference standard using the intersection of all DEG calls by edgeR, NOISeq, DEGseq, and DESeq2 using four MCF7 samples in two conditions (8 samples), while the prediction of single-subject DEGs(ss-DEGs) was conducted on independent sample pairs three times using DESeq (3 independent pairs). Note that the prediction method is not part of the reference standard construction to mitigate for analytic biases (Table 1). The analysis consists of constructing the reference standard first on the entire set of MCF7 gene product counts and second on the optimal region of concordance as identified by the Jaccard Indices. Then, using this robust reference standard identified by *referenceNof1*, an exemplar analysis in a hold-out pair of single-subject paired-transcriptomes, DESeq is used to identify altered genes and the results are compared against the reference standard. This process is repeated three times, once on each of the three hold-out sets (sss-DEGs). The average results across all three hold-out sets are presented in Table 3. For comparison, the results are shown for an equivalent DESeq analysis on the full, unfiltered hold-out sets.

### 2.9. ReferenceNof1 R Package

The code SamirRachidZaim/referenceNof1_study was re-packaged into a reproducible and shareable R-package format, available for installation on GitHub under the following repository: SamirRachidZaim/referenceNof1.

## 3. Results

### 3.1. Fold Change Region Analysis

We utilize a previously designed dataset comprising of repeated biological assays and transcriptomes of MCF7 breast cancer cell lines in two conditions (exposure or no exposure to estrogen). This is used to derive a reference standard to evaluate analytical methods deriving DEGs from one sample in each condition [4,12]. Concordances between conventional RNA-seq analytical methods for repeated measurements (Methods) applied to this MCF7 dataset were calculated and shown in Table 4. The calculated differentially expressed genes (DEGs) are filtered at different fold changes (FC). In low fold change regions, the highest agreement between analytical methods reached 50%, providing low trust across the competing signals detected across analytical methods. The analytical methods are not validating each other’s discoveries. However, as the FC effect size increases, the increasing FC regions demonstrate greater agreement across analytical methods, resulting in FC conditions enabling the evaluation of one method against another—eliminating isomorphic analytical biases. Table 4 numerically illustrates this trend, noting that the Jaccard Indices for the first pair of fold change regions is mostly near or at zero, except for a few combinations. In the absence of a framework or analytical method for resolving this conflict of signal, any study that chooses a particular analytical method risks to detect a non-robust signal beyond naïve reproducibility. At higher fold change regions above 1.2, there is no one “best” fold change region, supporting the idea that filtering FC≥1.3 may provide the most reproducible results from method to method for this specific dataset.

### 3.2. Combining Fold Change and Low-Expression Noise Reduction in Reference Standards

Combining gene expression levels (minimum expression cutoff; Figure 2 and Figure 3) and effect-size prefiltering results in a quasi-linear improvement in agreement. The experimental design allowed us to examine the marginal effects of increasing cutoffs for gene expression and effect size (see Figure 2 for Breast Cancer and Figure 3 in Yeast).

In the breast cancer study, the reference standards were constructed using four replicates. The bottom right portion of concordances in Figure 2 illustrates how all the analytical methods attained strong concordances due to the strictest thresholds (at least > 75% of all identified DEGs).

In the yeast study, smaller effect size thresholds were required for analytical methods to have complete agreement (see Figure 3). The methods appear to agree and produce concordant reference standards after imposing moderate (at least on our scale of parameters) fold change and expression cutoff values. It suggests perhaps that these analyses might benefit from developing a self-learning algorithm that finds the optimal cutoff values after conducting a grid search on the parameter space.

### 3.3. DESeq Example Analysis with Robust Reference Standards

Table 5 illustrates the calculated single-subject DEGs (ss-DEGs) from two samples (one in each condition, without replicate) from MCF breast cancer cells using DESeq. The discovered DEGs were evaluated against either a reference standard constructed from the intersection of DESeq2, edgeR, NOISeq, and DEGseq (Table 5, top row) or using the optimized reference standard using the proposed *referenceNof1* method (Table 5, bottom row). This experimental design using isogenic cells lines with replicates in two conditions enables validation of single-subject transcriptome analysis methods. This evaluation addresses the limitations highlighted in Table 1. The results in Table 5 indicate a substantial increase in accuracy in single-subject studies using single-subject DESeq taking one sample in each of the two MCF7 cell lines conditions without replication (average recall and precision shown as we obtained three measures of each for every reference standard). As shown in Figure 1, we took three distinct pairs of estrogen exposed and unexposed samples and calculated, for each pair, the ss-DEGs using DESeq as an exemplar method being applied to a clinical sample. Prior studies using DESeq in single-subject studies indicate a conservative prediction approach, producing few but highly precise DEG calls [12,14,15]. These results indicate a consistent operational characteristic as well as an improved region of algorithm accuracy.

## 4. Discussion, Limitations, and Future Studies

### 4.1. Discussion

We and others have proposed that while identifying altered DNA is possible using reference standards derived from populations, determining altered gene products (e.g., transcriptomes, proteomes and metabolomes) are better determined in isogenic conditions [2]. Indeed, identical twins sharing the same DNA but living in diametrically different environments (polar vs. artic) and having different dietary, sleeping, and exercise regimen may have normal, yet quite distinct, gene products in their cells. This motivated us and others to design analytical methods to determine personalized differentially expressed genes (DEGs) from two samples without replicates, each taken in a different condition. These approaches have been applied for (i) comparing a cancer transcriptome to a paired control tissue [6], and (ii) comparing peripheral blood mononucleocytes of a single subject either taken in two conditions (e.g., before and during therapy to predict response [26], separated in two petri dishes with one experimentally exposed to a virus vs. a control to determine ulterior hospitalizations in pediatric asthmatic subjects [26,27], etc.). While these single-subject study designs are economical and more informative than a single measure of the transcriptome, they remain difficult to evaluate. We have previously shown that these single-subject DEGs analytics designed for two paired samples can be better evaluated by using previous cell line datasets comprising of multiple replicates across both conditions. However, most conventional methods generated a conflicting discordant reference standard, motivating the current study.

Constructing a robust and reproducible reference standard should not be confounded with identifying or predicting DEGs for a gene-expression classifier. Since gold standards on an Omics scale are truly only available in simulation studies, we propose to call standards derived from Omics-scale analyses of biologic datasets reference standards. Therefore, a novel framework is required to address the difficulties to generate a reliable reference standard as highlighted in Table 1. The proposed framework provides an alternative reference standard construction that is robust against violations of statistical assumptions, resolves competing signal across analytical methods, produces accuracy beyond naïve reproducibility, and serves as a starting point for expanding robust reference standards in the other ‘omics. This proof-of-concept with transcriptomics provides encouraging preliminary results.

The modus operandi of developing a reference standard consisted of using a technique to build a reference standard (i.e, DEGseq) and measuring it against itself via some criterion (i.e., AUC). This provides naïve reproducibility that does not generalize across methods. Our proposed framework, *referenceNof1*, generates an unbiased and robust reference standard using concordance between heterogenic methods using transcripts. We note how filtering out noise (i.e., genes with small fold change) can have a drastic effect for improving on how to build reference standards as well as provides a framework that quantifies these differences and provides guidance on how to construct the reference standard for a biomarker analysis independent of the used technique. In addition, filtering out genes with low expression [23,24,25] improves the power and removes the noise in bioinformatics, therefore ensuring that uniform cutoffs across techniques improve their concordance, thus increasing their reliability. In addition, removing low expression and low effect-size genes may lose many genes, requiring one to control for false negative rates. This is where an ensemble-like approach (i.e., referenceNof1) controls for individual technique’s false negatives, as a “majority-vote” rule is robust to individual false negatives as long as the techniques can identify low effect-size genes. However, a more systemic approach is necessary when all techniques compared result in false negatives. Constructing methods-agnostic reference standards will only enable the community to continue improving the state of reproducibility in bioinformatics data analysis. Clearly, the solution range is dataset-specific as shown by the different concordances of the same methods applied to two different datasets (Figure 2 and Figure 3). This study suggests that a “stratification data analysis model” could be applied to determine the optimal gene expression cutoff required to obtain the desired concordance for each range of fold changes and the minimal fold change required to include results in the reference standard. For example, if a concordance of Jaccard Index > 75% for a simple majority of DEG methods is considered sufficient for generating a reference standard, then Figure 2 shows that an expression > 30 and FC > 1.3 meet these criteria. If the criteria were reduced to Jaccard Index > 50% and a simple majority vote, then the reference standard comprises the DEGs discovered at the union of [expression cutoff > 0 and 1.3 < FC < 1.5] and [expression cutoff > 30 and FC > 1.5]. Reference standards built for a single subject are isogenic by design. Since most of the publicly available human transcriptome datasets consist of measures for multiple subjects (heterogenic), we conducted the validation of our proposed methods using multiple measures in two experimental isogenic conditions in cell lines as proxy for a single-subject study. The example study with DESeq illustrates the added utility in creating robust reference standards. We have previously documented that some DEGs methods designed for comparisons requiring repeated measures of isogenic samples or many heterogeneric samples for each condition claim to be applicable to single-subject analyses of transcriptome methods, yet none had documented their validation [2]. Subsequently, Schurch et al. [16] and Liu et al. [13] validated NOISeq, DEGseq, DESeq2, and EdgeR using replicates of isogenic samples in two conditions conducted in MCF7 and yeast data, respectively; however, their evaluations were conducted using anticonservative designs generating analytical biases due to isomorphic evaluations (Table 1) as shown in our recent study [12].

### 4.2. Limitations and Future Studies

The *referenceNof1* algorithm provides tools to create robust reference standards in transcriptomic studies, however there are a number of opportunities to improve and extend this research. Three areas have been identified to extend the research and utility of *referenceNof*1 and are discussed below.

The first is to create a self-learning algorithm to expand the search space to identify global or local optimal parameters, rather than search strictly within the user’s predefined grid-search. As shown in Figure 2 and Figure 3, stricter cutoffs resulted in higher concordances in the breast cancer cell line dataset, whereas in the yeast dataset, the concordances followed a parabolic behavior, with initial increases and then a decrease in concordances. This limits the users to identify optimal solutions within their “user-defined” search space and suggests that there is not necessarily a universal effect-size “cutoff” after which methods agree, but rather data-driven fold change (FC) regions in which the agreements are maximized. Since these FC regions need to be identified on each dataset, we posit that a self-learning algorithm can be implemented to identify high-agreement regions with the goal of identifying the smallest thresholds that attain the maximal Jaccard Index to preserve the majority of the data.

Preliminary work in *referenceNof1* has been extended in this direction with future studies focused on fine-tuning and refining the approach to having a self-learning algorithm optimize the operating space to better guarantee biological validity for the statistical results. Currently, the search is designed to identify the minimal set of operating parameters to attain a certain Jaccard Index concordance using the intersection of techniques. This can be combined with including a “majority-voting” rule and generalize the voting scheme to allow greater flexibility.

Since signal can be unstable in single-subject studies, the second direction for future studies should consider incorporating additional scales of biology offers an opportunity to incorporate ontologies to stabilize the signal using pathways. As shown in [28], biological signal in heterogenic subjects can be unstable and expressed inconsistently across subjects, suggesting that alternatively it might be more effective to conduct pathway-level analyses and train pathway-level classifiers. Therefore, another direction to extend this strategy is to improve and refine the state of pathway-level reference standards by incorporating ontologies like Gene Ontology [29] and other network-analysis tools into building a robust and reproducible reference standard.

Finally, the third direction is to extend *referenceNof1* to single-subject studies of biomolecular pathway dynamics and into the other ‘omics. The current manuscript presents a framework for robust reference standards in transcriptomics. However, we and others have previously demonstrated the utility of single-subject analytics of gene products at ‘omics scale. For example, we have shown how to compare treated vs untreated peripheral blood mononucleocytes (PBMCs) using single-subject transcriptome analyses designs [26], as well as contrasting experimentally stimulated vs unstimulated PBMCs of a subject ex vivo using rhinovirus to predict hospitalization in asthmatic subjects [27], or comparing cancer vs. adjacent control tissue of a subject predicting response to therapy [6]. Therefore, future studies should consider extending the *referenceNof1* framework into the other ‘omics in order to further enable and strengthen single-subject studies across the ‘omics.

## 5. Conclusions

Reproducibility and accuracy are not only central to Omics studies but to precision medicine. Improving existing techniques and frameworks in single-subject studies empowers scientists to separate clinically relevant biomarkers from statistical artefacts. Transforming these initiatives into open-source software improves reproducibility and furthers the space of open precision medicine. Prior studies [2] illustrate how the unique challenges of single-subject analyses of transcriptomes in the absence of replicates remain challenging. However, we posit that an improvement in evaluation methods, as proposed here, provides the rigorous framework for assessing objectively ulterior proposed improvements. In addition, pathway-level single-subject studies of transcriptomes have been shown more accurate than gene product level ones [3], suggesting potential future pathway-level applications of the methods we proposed. This manuscript highlights four types of biases (Table 1) that confound results in both conventional analyses and single-subject studies’ clinical translation. The proposed *referenceNof1*, complementary to [12], follows a suite of recent work [9,12,28] in which we seek to address these challenges, resulting in a new framework for creating robust reference standards. We proposed, tested, and developed an open-source software using a single strategy that reduces two additional biases: (i) Statistical distribution bias and (ii) systematic bias from isomorphic evaluations (using the same analysis in the prediction and validation sets). Despite the specific challenges posed in single-subject studies, these advances create new opportunities to combine single-subject and conventional cohort studies. In this study, we demonstrated the utility of constructing more robust reference standards in single-subject transcriptomic studies. There are multiple directions to conduct future studies. One opportunity is a follow-up study that will extend these methods by incorporating ontologies to transform and aggregate gene-products into pathways and gene sets, which can construct pathway-based robust reference standards. An alternate avenue can be pursued by extending these tools into other ‘omics (i.e., proteome or metabolome). Finally, future studies will include a self-learning grid-search that identifies the optimal reference standard parameters. This manuscript expands upon recent work to addresses existing knowledge gaps and challenges in the single-subject domain to bring our tools, technology, and analyses closer to delivering the promise made by precision medicine: “the right treatment, for the right patient, at the right time.”

## Figures and Tables

**Table 1:** Current limitations with biased gold standards in transcriptomic gene expression in single-subject studies.

Issue	Description
Statistical assumptions bias	When conditions of applicability (e.g., homoscedasticity assumptions) of the theoretical distribution of the underlying analytics are overlooked and unapplicable, prioritized results contain biases (false positives and false negatives) inherent to modeling inadequacies.
Analytical bias and systematic errors	Studies that use the same analytical method for the prediction calculation as for the reference standard construction incorrectly confirm systematic errors leading to analytical biases. For example, creating a reference standard with the same analytical method (isomorphic evaluation) as the one generating predictions can lead to “naive replication” of results comprising both true and false positives (biased systematic artefacts of a specific analytical method). Isomorphic evaluations in Omics analyses are anti-conservative by design.
Conflicting biomarker predictions in a single subject	Single-subject studies lack references by design: what happens when analytical method A and analytical method B disagree on a gene’s significance? Is gene *x* really significant? There is a lack of accuracy framework for evaluating and resolving conflicting signal stemming from distinct DEG analytics in a single-subject analysis.
Dataset dependency biases	Reusing part of the reference standard data for generating predictions creates dependencies, an evaluation framework problem observed more frequently in statistical evaluation of isogenic data [11,12].

**Table 2:** The two isogenic datasets include a single individual’s gene expression dataset with 7 biological replicates while the second dataset has 48 wild-type and mutant biological replicates.

Dataset	Samples	Genome Size (# Genes)	Access to Data
MCF7 Breast Cancer [13]	7	~20,000	GEO: GSE51403
Yeast [16]	48	~7000	Github: bartongroup/profDGE48

**Table 3:** Parameter settings in experimental design.

Parameter	Values
Fold change window	[1–1.1], [1.1–1.2], [1.2–1.3], [1.3–1.5], [1.5–∞]
Low expression cutoff	0, 5, 10, 20, 30, 50

**Table 4:** Concordance between analytic methods of RNA sequencing according to ranges of gene expression fold changes (FC) between two conditions. The low concordance observed in most FC ranges illustrate the “analytical bias of methods” described in Table 1. Indeed, if the same method is used for prediction in one dataset and validation in a distinct dataset (isomorphic evaluation), the evaluation is considered anticonservative as it measures the reproducibility of true positive and false positive results (analytical biases) rather than a measure of accuracy. In addition, the table results illustrate the difficulty to create a conservative reference standard for which the analytical method would be independent from the predictive method (heteromorphic evaluation); there is no single method that would be the best choice “a priori” to evaluate a new method. Legend: Since the Jaccard Index is symmetric, for any two techniques, we present the Jaccard Indices for the $\left(\begin{array}{c} 5\\ 2 \end{array}\right)=10$ total possible pairwise combinations between the five analytical methods evaluated across the different fold change regions. DEGs were calculated using five repeated samples of MCF7 breast cancer cell lines exposed to estrogen and five unexposed samples. The high concordance for each pairwise comparison is bolded (Jaccard Index > 0.6) and shown in a larger font. (Legend: In the manuscript and figure, we represent FC for upregulated genes and 1/FC for downregulated genes).

Analytical Method A	Analytical Method B	1 < FC < 1.1 (~85 DEGs)	1.1 < FC < 1.2 (~175 DEGs)	1.2 < FC < 1.3 (~700 DEGs)	1.3 < FC < 1.5 (~1100 DEGs)	1.5 < FC < ∞ (~365 DEGs)
NOISeq	edgeR	0.500	0.333	**0.885**	**0.819**	**0.631**
NOISeq	DESeq	0	0	**0.814**	**0.747**	0.586
NOISeq	DESeq2	0.005	0.002	0.311	0.436	0.372
NOISeq	DEGseq	0	0	0.355	0.569	**0.672**
edgeR	DESeq	0	0	**0.902**	**0.868**	**0.795**
edgeR	DESeq2	0.002	0	0.329	0.468	0.515
edgeR	DEGseq	0	0	0.387	0.558	**0.658**
DESeq	DESeq2	0	0.076	0.332	0.457	0.489
DESeq	DEGseq	0	0.285	0.415	0.555	**0.661**
DESeq2	DEGseq	0.005	0.135	0.452	**0.654**	0.450

**Table 5:** **Single-subject DEGs predictions evaluated by conventional methods and *refereneNof1*.** In order to simulate transcriptomic data from a single patient, single-subject DEGs were calculated by the DESeq method from two samples (MCF7 cell vs. MCF7 exposed to estrogen) and evaluated against two reference standards derived from Y samples in each condition (2Y samples total). The two reference standards constructed in this exemplary study illustrate the increases in accuracy provided by the proposed *referenceNof1* method to increase the agreement between DEGs methods used for as a reference and mitigate analytical biases from isomorphic evaluations. The optimal region identified by the *referenceNof1* algorithm resulted in a DESeq prediction set with a substantially higher precision with a slightly higher recall. (Legend: In the manuscript and figure, we represent FC for upregulated genes and 1/FC for downregulated genes).

		Predictions of ss-DEGs Calculate by DESeq	
Reference Standard Construction	References True DEGs (*False= Remaining Transcripts*)	Average Precision	Average Recall
Intersection of DEGs between methods *	522 (*16,625*)	0.57	0.08
Majority vote of DEGs between methods *	1424 (*15,723*)	0.77	0.04
*referenceNof1* applied to intersection of DEGs * between methods	165 (*16,982*)	0.70	0.12
*referenceNof1* applied to majority vote of DEGs * between methods	406 (*16,741*)	0.85	0.06

* calculated by DESeq2, EdgeR, NOISeq, DEGseq NOT using DESeq to avoid analytical biases.

## Data Availability

The datasets used in this study are publicly available. Their access details are provided in Table 2.

## Supplementary Materials

The following are available online at https://www.mdpi.com/2075-4426/11/1/24/s1. Table S1: Replicated DEG Methods for Single-Subject Studies and their previous validations, Table S2: Example of building a Jaccard Index matrix to optimize the Reference Standard Optimization.

## Mathematical Notation

**Notation & Variable**	**Variable Description**	**Equation**
A_k_ and B_k_	Gene product expression of gene *k* in conditions *A* and *B*	Figure 1
FC	Fold change: we represent FC for upregulated genes and 1/FC for downregulated genes.”)	Equation (1)
Jaccard Index = $\frac{\left|M\cap N\right|}{\left|M\cup N\right|}$	Jaccard index is the ratio of significant gene products in common between results derived from analytical methods *M* and *N* divided by the union of these sets	Equation (2)
M∩N	Intersection of sets *M* and *N*	Equation (2)
M∪N	Union of *M* and *N*	Equation (2)
|*M*|	Cardinality or size of set *M*	Equation (2)
*Ri*	Region i, portion of the transcriptome resulting from the filters and cutoffs selected in *referenceNof1*	Algorithm 1
*JI _(M,N),Ri_*	The Jaccard index between analytics methods M and N for genes in region *Ri*	Algorithm 1

## Acronyms and Abbreviations

**Abbreviation**	**Name**
DEGs	Differentially expressed genes
SSS	single-subject studies
FC	Fold change for upregulated genes and 1/Fold change for downregulated genes
NHST	Null Hypothesis Significance Testing
