# Supplementary material for: Personalized beyond Precision: Designing Unbiased Gold Standards to Improve Single-Subject Studies of Personal Genome Dynamics from Gene Products
Source: J Pers Med. 2020 Dec 31;11(1):24. doi: 10.3390/jpm11010024 (PMC7823282; doi:10.3390/jpm11010024)
Supplement: Supplementary file 1 [file jpm-11-00024-s001.pdf]

Supplementary Material:

Supplementary Table 1. Replicated DEG Methods for Single-Subject Studies and their previous validations.

Method	Distribution assumptions	Experimental design & parameter settings	P-value
edgeR	Negative Binomial	"genetically identical model organisms"	✓
DESeq	Negative Binomial	*estimateDispersions* function, the method parameter is set to 'per-condition'	✓
DESeq2	Negative Binomial	Default parameters	✓
DEGseq	Binomial	Default parameters	✓
NOISeq[1]	Non-parametric	Noiseqbio set to default parameters	✓

Supplementary Table 2. Example of building a Jaccard Index matrix to optimize the Reference Standard Optimization. An example Jaccard Index matrix where once all techniques have produced a list of DEG calls, then the pairwise Jaccard Index can be calculated as illustrated in Algorithm 1, resulting in a Jaccard Index matrix below. Then, for each set of parameter combinations (one expression cutoff value, one fold-change region), a Jaccard concordance matrix is constructed, and summarized using the median value. Then all medians are compared to identify the best parameter configuration to identify the region for constructing the optimal, most robust reference standard.

	NOISeq	edgeR	DESeq	DESeq2	DEGseq
NOISeq	1	0.8	0.71	0.2	0.17
edgeR	0.8	1	0.57	0.21	0.2
DESeq	0.71	0.57	1	0.25	0.29
DESeq2	0.2	0.21	0.25	1	0.15
DEGseq	0.17	0.2	0.29	0.15	1

[1] NOISeq-Bio was used to construct the reference standard, while NOISeq-sim was used in the single-subject prediction sets.
